# Structure of Putrescine Aminotransferase from *Escherichia coli* Provides Insights into the Substrate Specificity among Class III Aminotransferases

**DOI:** 10.1371/journal.pone.0113212

**Published:** 2014-11-25

**Authors:** Hyung Jin Cha, Jae-Hee Jeong, Catleya Rojviriya, Yeon-Gil Kim

**Affiliations:** Pohang Accelerator Laboratory, Pohang University of Science and Technology, Pohang, Korea; Russian Academy of Sciences, Institute for Biological Instrumentation, Russian Federation

## Abstract

YgjG is a putrescine aminotransferase enzyme that transfers amino groups from compounds with terminal primary amines to compounds with an aldehyde group using pyridoxal-5′-phosphate (PLP) as a cofactor. Previous biochemical data show that the enzyme prefers primary diamines, such as putrescine, over ornithine as a substrate. To better understand the enzyme's substrate specificity, crystal structures of YgjG from *Escherichia coli* were determined at 2.3 and 2.1 Å resolutions for the free and putrescine-bound enzymes, respectively. Sequence and structural analyses revealed that YgjG forms a dimer that adopts a class III PLP-dependent aminotransferase fold. A structural comparison between YgjG and other class III aminotransferases revealed that their structures are similar. However, YgjG has an additional N-terminal helical structure that partially contributes to a dimeric interaction with the other subunit via a helix-helix interaction. Interestingly, the YgjG substrate-binding site entrance size and charge distribution are smaller and more hydrophobic than other class III aminotransferases, which suggest that YgjG has a unique substrate binding site that could accommodate primary aliphatic diamine substrates, including putrescine. The YgjG crystal structures provide structural clues to putrescine aminotransferase substrate specificity and binding.

## Introduction

Polyamines, including putrescine, spermidine and spermine, are aliphatic amines with two or more amino groups and are distributed widely among prokaryotic and eukaryotic cells [1], [2]. Polyamines play an important role in regulating various cellular functions, such as protein synthesis as well as cell proliferation and development [1]. Polyamine concentrations increase in proliferating cells, such as cancer cells and bacterial cells during exponential growth [3], [4]. However, over-accumulation of polyamines can induce apoptosis and inhibit cell growth [5], [6]. Therefore, polyamine levels in the cell need to be regulated by synthesis, degradation, uptake and excretion systems [7].

The major *Escherichia coli* polyamine, putrescine, is produced either from ornithine by the ornithine decarboxylase enzyme or from arginine by the arginine decarboxylase and agmatinase enzymes [8], [9]. Putrescine is degraded through two pathways, the Puu and aminotransferase pathways. In the Puu pathway, putrescine is degraded to succinic semialdehyde, which is a succinate precursor, via γ-glutamyl intermediates [10], [11]. In the aminotransferase pathway, putrescine is metabolized to γ-aminobutyric acid (GABA) without γ-glutamylation by the YgjG putrescine aminotransferase and the YdcW γ-aminobutyraldehyde dehydrogenase [12], [13].

YgjG is a putrescine aminotransferase (PATase, EC 2.6.1.82). Based on its amino acid sequence alignment, it belongs to the class III pyrodoxal-5′-phosphate (PLP)-dependent aminotransferase family, which include ornithine aminotransferase (OAT) and GABA aminotransferase (GABA-AT) [12], [14]. Similar to many other PLP-dependent aminotransferases, PATase is expected to undergo two half-reactions (Figure S1). In the first half-reaction, putrescine is converted to *γ*-aminobutyraldehyde, and the putrescine amino group is transferred to the PLP cofactor to yield pyridoxamine phosphate (PMP); this *γ*-aminobutyraldehyde is spontaneously cyclized to Δ^1^-pyrroline [11]. In the second half-reaction, the PMP amino group is transferred to the α-ketoglutarate to yield L-glutamate and regenerate PLP.

Previous biochemical studies have shown that the YgjG enzyme prefers primary aliphatic diamines, such as putrescine, over ornithine or GABA as a substrate [12], [15], which suggests that its structure could differ from other class III aminotransferases, such as OAT and GATA-AT. In this study, we determined the crystal structures of YgjG from *E. coli* in free and putrescine-bound states at 2.3 and 2.1 Å resolutions, respectively, to investigate the structural basis of YgjG substrate specificity. This is the first structure of an aminotransferase for polyamines, including putrescine. When the overall YgjG structure was compared with other class III aminotransferases, its structure was similar to the other class III aminotransferases. However, YgjG has a smaller and more hydrophobic entrance to the substrate-binding cleft compared with other class III aminotransferases, which suggests that it could accommodate primary aliphatic diamine substrates, such as putrescine, rather than the bulky and hydrophilic ornithine. Our findings will aid in better understanding the different substrate specificities among aminotransferases.

## Materials and Methods

### YgjG purification

YgjG from *E. coli* was expressed and purified as described previously [16], [17]. Briefly, YgjG was expressed as a C-terminal His6-tagged fusion protein in the *E. coli* B834(DE3) strain (Novagen) and purified using a Ni-NTA resin-based chromatography column. The protein sample was further purified though ion-exchange chromatography followed by size-exclusion chromatography equilibrated in 10 mM Tris-HCl (pH 8.0) and 100 mM NaCl.

### Crystallization and structure determination

Native YgjG crystals were generated using the hanging-drop vapor-diffusion method at 22°C by mixing and equilibrating 2 µl each of the protein solution and a precipitant solution containing 0.1 mM n-dodecyl-N, N-dimethylglycine, 0.2 M sodium formate, 15% PEG 3350, and 0.1 M HEPES (pH 7.5) as described previously [16]. The putrescine-bound structure was generated using the soaking method. For data collection, the crystals were briefly immersed in the same precipitant containing an additional 15% glycerol as a cryoprotectant and immediately flash-cooled in a 100 K nitrogen gas stream. Diffraction data were collected using an ADSC Q315r CCD detector on beamline 5C at Pohang Accelerator Laboratory (PAL), Pohang, Korea. A total of 240 images were collected with an oscillation of 1° and a crystal-to-detector distance of 200 mm. The crystal was exposed for 1 s per image. The data were indexed, integrated and scaled using the HKL-2000 software package [18]. The YgjG structures were solved by using molecular replacement [19]. The structure of acetylornithine aminotransferase (AcOAT) from *Thermus thermophilus* (PDB code 1VEF) was used as a search model to solve the YgjG crystal structures. Model building and refinement were performed using Coot [20] and the Phenix package [21], respectively. The x-ray diffraction and structure refinement statistics are summarized in Table 1.

**Table 1:** Structure refinement statistics.

Data Collection	Native YgjG	YgjG-putrescine
Space group	*P*2_1_2_1_2_1_	*P*2_1_2_1_2_1_
Unit cell dimensions		
a, b, c (Å)	120.9, 129.3, 131.1	121.1, 129.5, 131.3
α, β, γ (°)	90, 90, 90	90, 90, 90
Resolution (Å)	30.0–2.3 (2.34–2.3)	50.0–2.1 (2.1–2.09)
*R*_merge_ a	10.5 (36.3)^b^	8.1 (31.7)
*I*/σ(*I*)	13.4 (3.0)	12.6 (2.4)
Completeness (%)	98.3 (96.9)	89.0 (81.9)
Redundancy	6.1 (4.3)	4.8 (2.8)
**Refinement**		
Resolution (Å)	29.7–2.3	36.3–2.1
No. of reflections	91029	123102
*R*_work_ c/*R*_free_	18.1/23.3	19.1/24.5
No. atoms		
Protein	13731	13759
Ligand	76	123
Water	351	513
R.m.s deviations		
Bond lengths (Å)	0.009	0.008
Bond angles (°)	1.136	1.172
Average B-values (Å^2^)	28.9	26.8
Ramachandran plot (%)		
Most favored region	89.9	89
Additionally allowed	9.6	10.3
Generally allowed	0.3	0.4

a *R*_merge_  =  Σ |*I*_obs_ - *I*_avg_|/*I*_obs_, where *I*_obs_ is the observed intensity of individual reflection and *I*_avg_ is average over symmetry equivalents.

b The numbers in parentheses are statistics from the highest resolution shell.

c *R*_work_  =  Σ ||*F*_o_| - |*F*_c_||/Σ |*F*_o_|, where |*F*_o_| and |*F*_c_| are the observed and calculated structure factor amplitudes, respectively. *R*_free_ was calculated with 5% of the data.

Stereochemical analyses of the free and putrescine-bound structures were performed using the program PROCHECK [22]. The atomic coordinates and structure factors are deposited in the Protein Data Bank with the codes 4UOY and 4UOX for the free and putrescine-bound YgjG, respectively.

### Thermal denaturation studies

The thermal denaturation experiments were performed using a Jasco J-810 spectropolarimeter equipped with a peltier temperature control system, as described previously [23]. Protein samples were prepared in 20 mM Tris (pH 8.0) in a 2 mm path length cuvette. The samples were heated from 25 to 105°C at rate of 1°C/min. Circular dichroism signals at 222 nm were recorded at 1°C increments.

### Structure analysis

The structures were superimposed using the Superpose program in the CCP4 suite [24]. The dimer interface was analyzed by using the Protein Interfaces, Surfaces and Assemblies (PISA) server [25]. Interactions between the YgjG and PLP cofactor residues were analyzed using the Contact program from the CCP4 suite [24]. Structure-based multiple sequence alignments were performed using the programs MultAlin [26] and ESPript [27]. All structural figures were generated using Pymol (http://www.pymol.org).

## Results and Discussion

### Overall structure

The native YgjG crystal structure was determined at the resolution 2.3 Å through molecular replacement and refined to the R_work_ and R_free_ factors 18.1% and 23.3%, respectively. The structure refinement statistics are summarized in Table 1. All residues in the two subunits, except for Lys300, are in the allowed and favorable regions of the Ramachandran plot, as defined by PROCHECK [22]. The electron density for the seven N-terminal and one C-terminal residues (including the Hi-tag) was not visible in the 2*F*_o_–*F*_c_ electron density map; therefore, they were not included in the final model.

**Figure 1:**
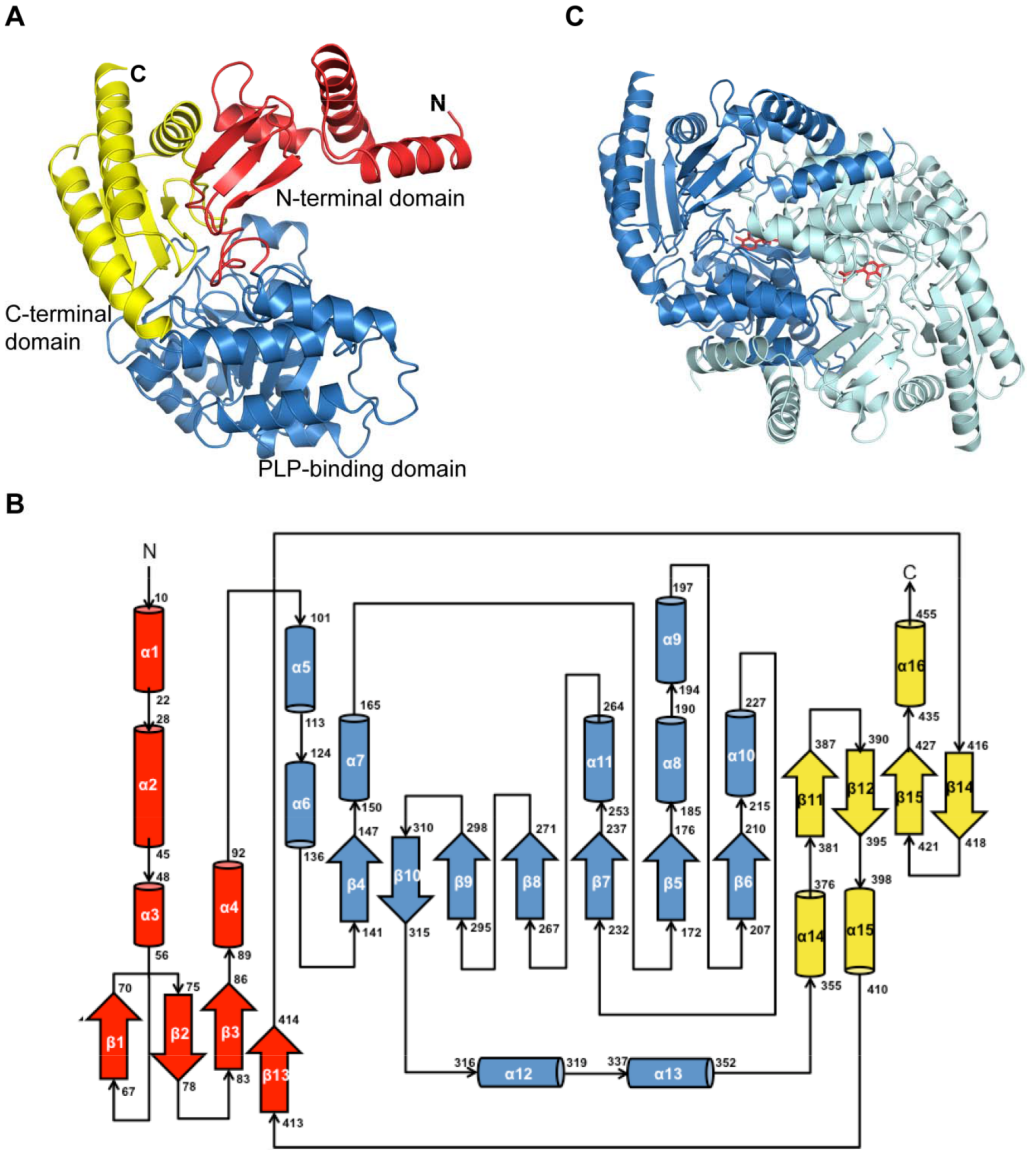
Overall YgjG structure. (A) Ribbon diagram of the YgjG monomer. The N-terminal domain, large PLP-binding domain and C-terminal domain are colored in red, sky blue and yellow, respectively. (B) YgjG topology diagram. The α-helices and the β-strands are represented as cylinders and arrows, respectively, and colored as in (A). (C) Ribbon diagram of the YgjG dimer. Subunits A and B are colored in sky blue and cyan, respectively. The PLP cofactor is shown as a stick model.

As shown in Figure 1A, the overall YgjG monomer fold is similar to those of other class III PLP-dependent aminotransferases [28]. Figure 1B shows the YgjG monomer topology. The monomer comprises an N-terminal domain, a large PLP-binding domain, and a C-terminal domain. The N-terminal domain (residues 1-100) contains four α-helices and a three-stranded antiparallel β-sheet. The large PLP-binding domain (residues 101–353) comprised a central seven stranded β-sheet surrounded by nine α-helices. The C-terminal domain (354–455) is composed of a four-stranded antiparallel β-sheet surrounded by three α-helices.

The crystallographic asymmetric unit contained four YgjG molecules that formed two dimers (Figure 1C). The dimer formed extensive contacts between the two subunits. The buried surface area between the two subunits was 6,167 Å^2^, which corresponds to 30.2% of the total solvent accessible area. It is mainly composed of polar interactions, including two pairs of salt bridge interactions, Glu44/Lys142 and Glu120/Arg54, and numerous hydrogen bonds along the interface (Table S1). Consistent with this result, the previous data from size exclusion chromatography revealed that YgjG forms a dimer in solution [16]. Therefore, YgjG could function as a dimer under physiological conditions, similar to other aminotransferases [29], [30].

### PLP-binding site

**Figure 2:**
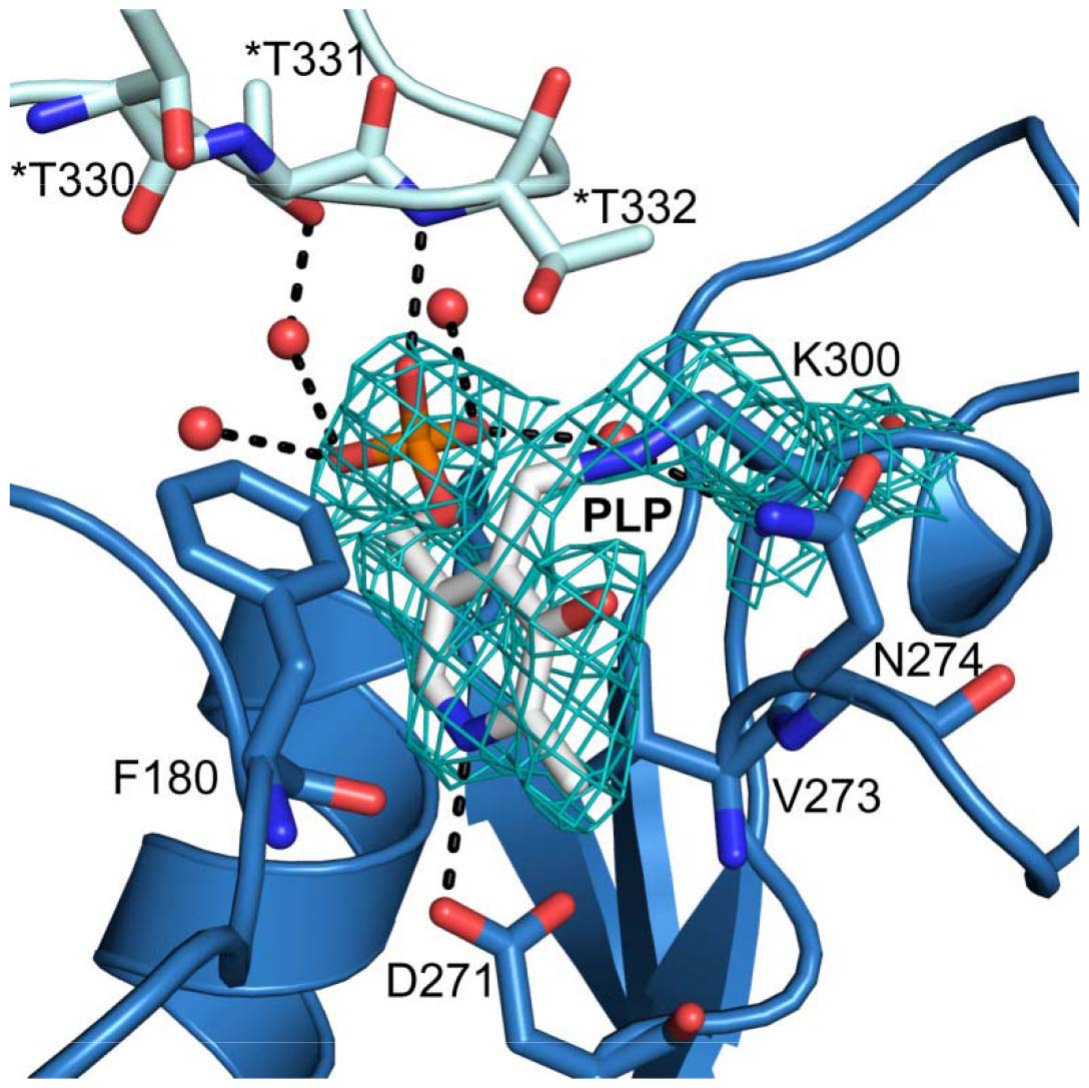
Closed-up view of the YgjG PLP-binding site. The residues interacting with PLP are represented as a stick model. The water molecules are shown as red spheres, and the hydrogen bonds are shown as black dashed lines. The * indicates a residue from the neighboring subunit.

**Figure 3:**
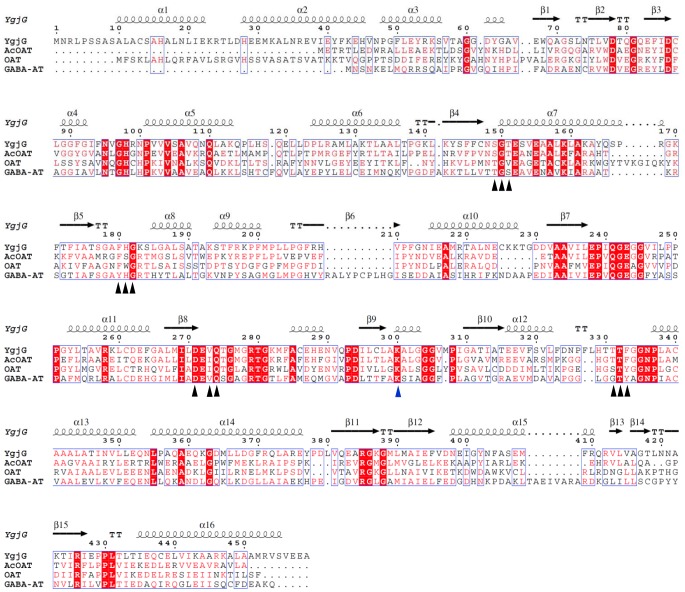
Structure-based sequence alignment of YgjG, AcOAT (PDB code 1VEF), OAT (PDB code 2OAT), and GABA-AT (PDB code 1SFF). The amino acid numbering at the top of the alignment is for *E. coli* YgjG. The residues involved in the PLP-binding sites are indicated by black triangles, and the conserved lysine residues are indicated by a blue triangle. The white letters on red background indicate fully conserved residues, while the red letters on white background indicate partially conserved residues. The secondary structural elements depicted at the top of the alignment correspond to those in YgjG. The structure-based sequence alignment figure was generated using ESPript [27].

The YgjG active site is at the dimeric interface and includes the residues from both subunits. The cofactor PLP is located at the bottom of the active site and is involved in multiple interactions (Figure 2). The residues that compose the PLP binding site are highly conserved according to the sequence alignment (Figure 3). The electron density for the cofactor clearly shows that PLP is covalently linked to the strictly conserved lysine residue (Lys300) via a Schiff base linkage (Figure 2). The PLP phosphate group interacts with four water molecules, Thr151, Gly150 and Thr332* (the asterisk indicating the other subunit) through hydrogen bonds. Similar to other class III aminotransferases [31], the Asp271 side chain is hydrogen bonded to the cofactor pyridine nitrogen. The PLP pyridine ring is sandwiched between the Phe180 and Val273 side chains. Overall, the binding pattern of PLP in YgjG is similar to other class III aminotransferases.

### Putrescine binding

**Figure 4:**
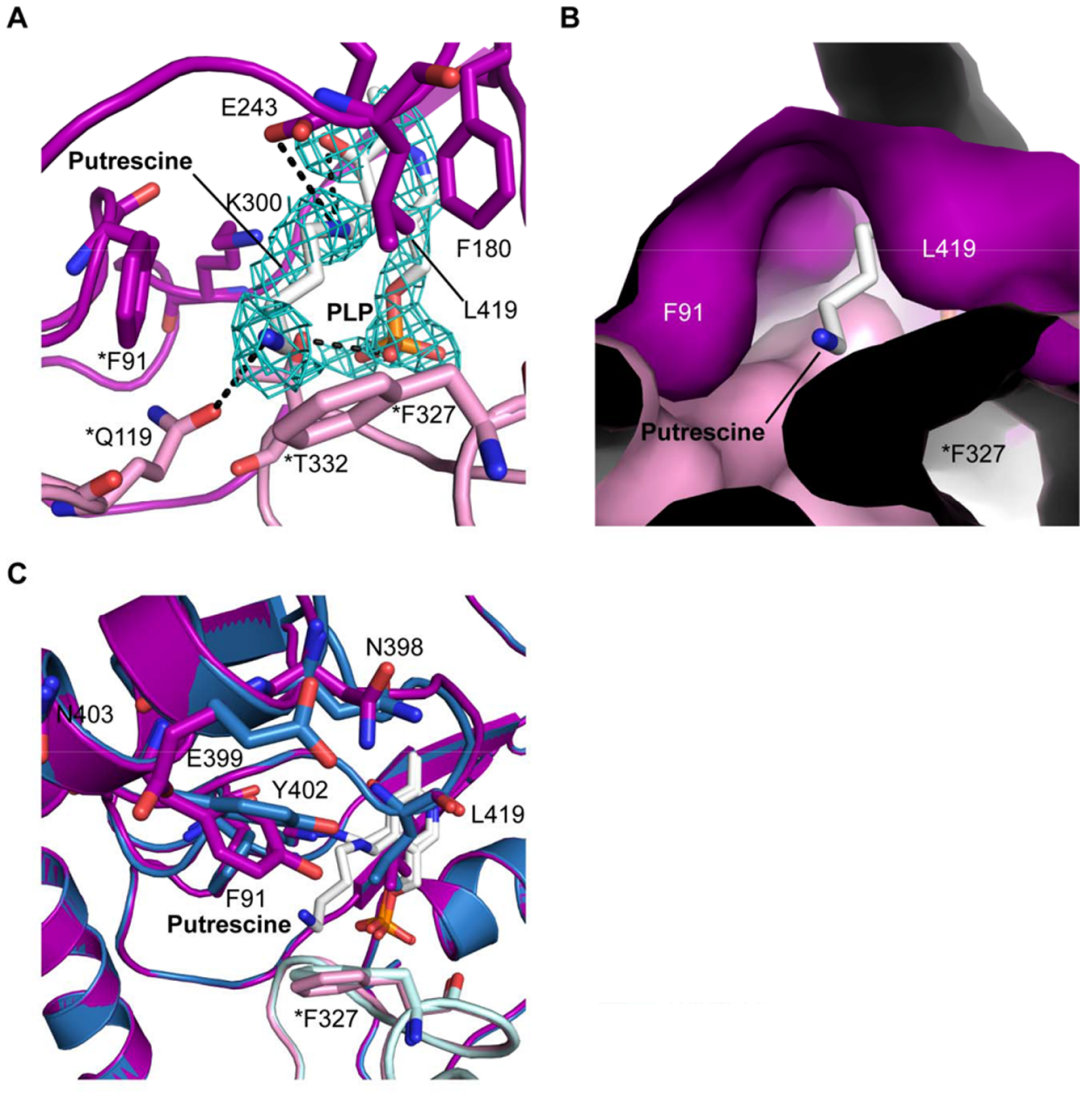
Putrescine binding to YgjG. (A) The 2*F*_o_–*F*_c_ electron density map is contoured at 1 sigma. The putrescine, PLP and active-site residues are represented as stick models. The hydrogen bonds are shown as dashed lines. (B) Surface representation of the substrate-binding cleft. Phe91, Leu419 and Phe327* occupy a large portion of the substrate-binding site entrance in YgjG. (C) Superposition of free YgjG and YgjG-putrescine complex. The side-chain movement of Tyr402 partially covers the active site.

The structure of YgjG in complex with PLP and putrescine was determined at 2.1 Å resolution. Figure 4A presents the bound putrescine electron density map. The putrescine electron density was observed in three of the four subunits in the asymmetric unit. The putrescine amino group bound the PLP at two of the three active sites. The covalent bond between PLP and Lys300 was broken, and the Lys300 side chain was displaced distal to the cofactor. The weak putrescine electron density could be explained by partial occupancy in the binding site and/or substrate flexibility.

Putrescine binding to YgjG is mediated by a number of polar and hydrophobic interactions (Figure 4A). The substrate N1 nitrogen atom forms hydrogen bonds with the Glu243 OE1 oxygen atom and the PLP O3 oxygen atom. The substrate N2 nitrogen atom was stabilized by a hydrogen bond with Gln119*. The putrescine was also surrounded by several residues, including Phe91, Phe180, Leu419 and Thr332*, which contributed to hydrophobic interactions with the substrate. Among these residues, the two hydrophobic residues (Phe91 and Leu419) together with Phe327* form a large portion of the substrate-binding cleft entrance (Figure 4B).

A structural comparison between the putrescine-bound and free forms of YgjG was performed to investigate whether PATase shows significant changes at the domain level, as observed in aspartate aminotransferase [32]. The overall structure of YgjG in complex with putrescine was almost identical to the unbound YgjG (r.m.s. deviations value 0.34 Å over 452 Cα atoms), which indicates no significant movement at the domain level upon substrate binding, in contrast to aspartate aminotransferase. Similar to YgjG, other class III aminotransferases, such as OAT, have been reported to undergo no significant domain closure when they bind their substrates [33]. However, several local conformational changes were observed in YgjG. In particular, the Tyr402 side-chain movement partially covered the substrate-binding site (Figure 4C); this movement could shield the hydrophobic binding site from solvent exposure.

### Comparison with other class III aminotransferases

**Figure 5:**
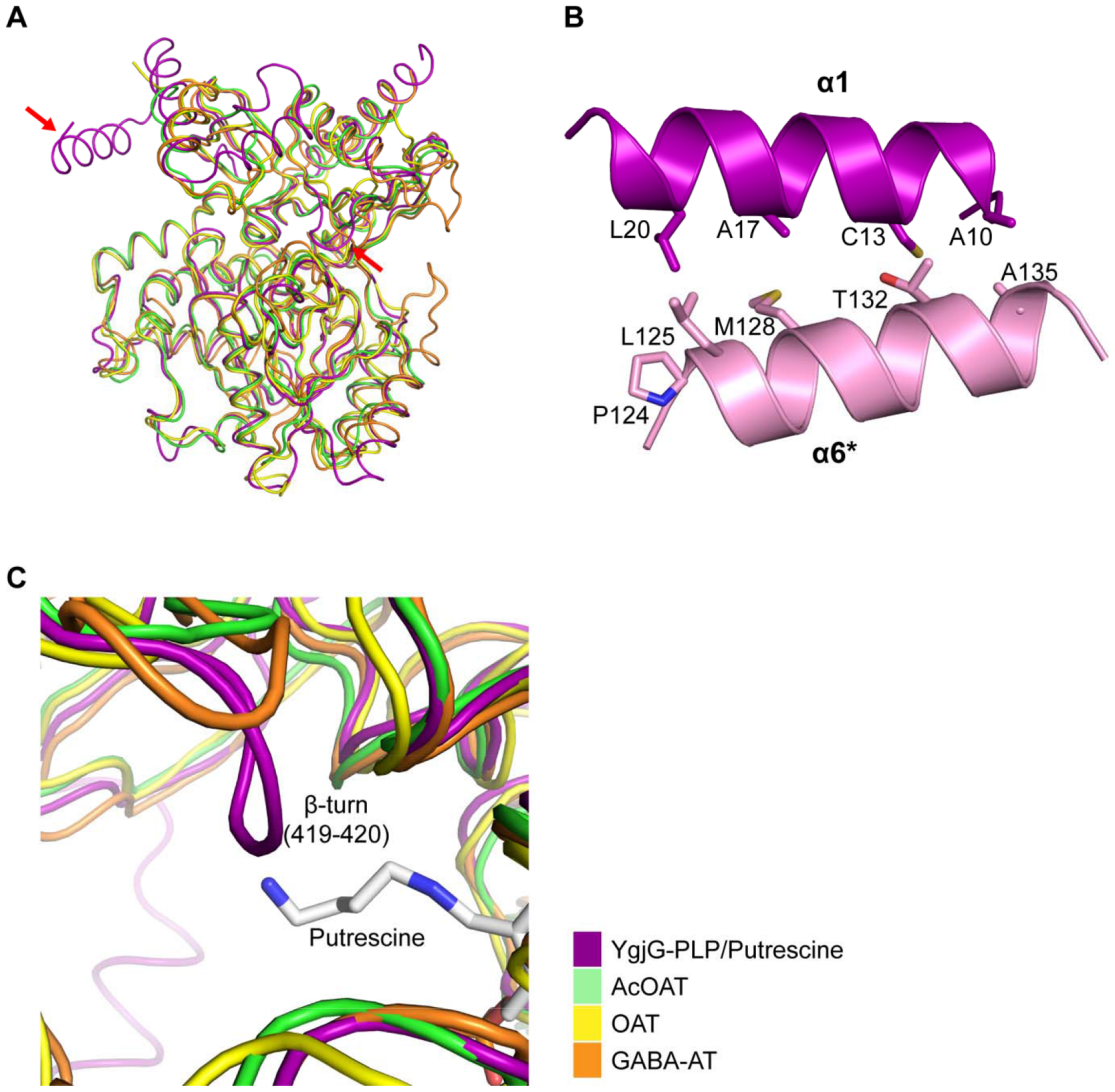
Comparison of YgjG and its structural homologs. (A) Superposition of the Cα trace for the YgjG monomer (magenta) with traces for AcOAT (green, PDB code 1VEF), OAT (yellow, PDB code 2OAT) and GABA-AT (orange, PDB code 1SFF). (B) Ribbon diagram of α-helix (α1) (magenta) and α-helix (α6) of the adjacent subunit (pink) in YgjG. Side chains of residues are shown in stick representation and are labeled. (C) When the YgjG Cα trace was superposed onto the AcOAT, OAT and GABA traces, the tight turn (residues 419–420) in YgjG protruded into the active-site cleft in contrast to its homologs. The Cα traces for YgjG and its structural homologs are colored as in Figure 3A.

Figure 5A shows overlays of the Cα traces for YgjG and other class III aminotransferases. Although the overall structures of these proteins are similar, certain local structural differences were also observed. The most remarkable difference is that YgjG has an additional N-terminal helix (α1) compared with its structural homologs. The α-helix (α1) (residues 10–22) in subunit A interacts with an α-helix (α6) (residues 124–136) in subunit B though a helix-helix interaction (Figure 5B). Due to the additional N-terminal helical structure, the dimeric interaction surface area of YgjG was greater than in other class III aminotransferases, such as OAT (Table S2). The increased interactions with the neighboring subunit might lead to a more stable dimer. Consistent with this structural analysis, an YgjG deletion mutant lacking an N-terminal region (1-30) showed a significant change in the thermal stability. Thermal denaturation data showed that the midpoint melting temperature (*T*_m_) value of deletion mutant protein (*T*_m_ = 48.6±0.3°C) was significantly lower than that of full-length YgjG (*T*_m_ = 81.4±1.0°C). Our structural and thermal analyses indicate that the additional N-terminal helical region in YgjG could contribue to additional stabilization through increased interactions with the adjacent monomer.

A prominent difference was also observed near the active site. Unlike the highly conserved PLP-binding site, the substrate-binding site was not well-conserved between YgjG and its structural homologs. When the YgjG active site was superimposed onto other class III aminotransferases, a tight turn (residues 419–420) in YgjG protruded into the active-site cavity, which is distinct from other class III aminotransferases (Figure 5C). In particular, the Leu419 side chain in the turn protruded into the active-site cleft, which suggests that it could prevent substrate access to the active site. YgjG also has a bulky residue (Phe327) protruding into the substrate-binding site. The bulky side chains around the active-site entrance might not allow entrance of the larger substrates, such as ornithine. Consistent with this result, previous studies show that YgjG has high activity for primary aliphatic diamines, such as putrescine, but low activity for bulky ornithine [12], [15]. Therefore, our data suggest that the smaller active site entrance in YgjG could be ideal for accommodating putrescine, but not bulky ornithine.

### Structural insights into substrate specificity

**Figure 6:**
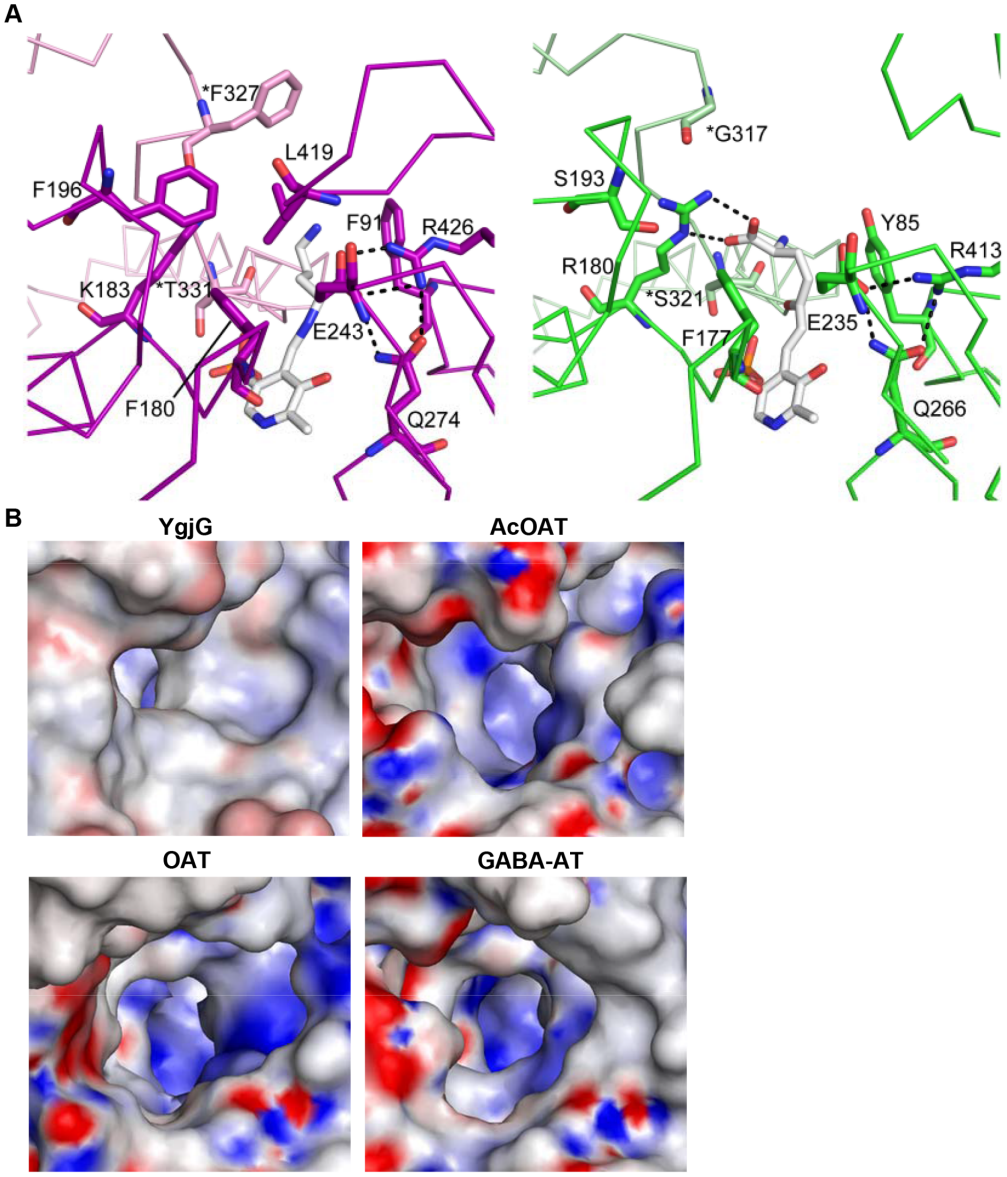
Comparison of substrate-binding sites. (A) Comparison of the substrate-binding sites in YgjG (in magenta) and OAT (green). The substrate-binding site of YgjG was in complex with the covalent adduct of PLP and putrescine. The substrate-binding site of OAT was in complex with a bound cofactor-inhibitor adduct (FMP). The PLP-putrescine adduct and FMP are shown as a stick model. Potential hydrogen bonds are shown as dashed lines. (B) Electrostatic potential surface representations of the substrate-binding site entrance in YgjG and its homologs. The YgjG entrance region is more hydrophobic than in AcOAT, OAT and GABA-AT.

Aminotransferase substrate-binding sites are characterized by dual substrate recognition [34]. Reports show that YgjG displays dual substrate specificity for putrescine or cadaverine as the amino donor and α-ketoglutarate as the amino receptor [12]. Like YgjG, most class III aminotransferases prefer α-ketoglutarate as the amino receptor in the second half-reaction [31]. Previous studies on the OAT structure [33] suggest that the Glu235-Arg413 ion pair opening could be required to interact with the α-ketoglutarate α-carboxylate group. Sequence and structure analyses show that the functionally important residues among the residues likely to interact with putrescine were sequentially and spatially conserved in the active sites (Figures 3 and 6A). In addition to the catalytic residue Lys300, the three residues (Glu243, Gln274 and Arg426) were strictly conserved. In YgjG, the residues Glu243 and Arg426 correspond to E235 and Arg413 in OAT, respectively (Figures 6A and 6B). Conservation of the glutamate-arginine ion pair suggests that YgjG could share a common mechanism in the second half-reaction with other class III aminotransferases.

In contrast, the YgjG substrate specificity in the first half-reaction differed from AcOAT, OAT and GABA-AT. The non-conserved residues near the active site could be important for the different substrate specificities in the first half-reaction among aminotransferases. There are two prominent non-conserved regions near the YgjG substrate-binding site: a Lys183 residue and a non-conserved region around the substrate-binding cleft entrance. Interestingly, YgjG has a lysine residue (Lys183) instead of the conserved arginine residue in AcOAT, OAT and GABA-AT. Acetylornithine, ornithine and GABA have a carboxyl group, whereas putrescine only contains aliphatic linear carbon atoms in its backbone chain. Reports show that the arginine residue in AcOAT, OAT and GABA-AT forms a strong salt bridge with the carboxyl group of their substrates [33], [35]. In OAT, the Arg180 residue was involved in positioning its substrate through interactions with the substrate analog carboxylate group (Figure 6B) [33]. The presence of Lys183 in this posision of the corresponding arginine in AcOAT, OAT and GABA-AT could be a reason for the low substrate activity of YgjG for the substrates with the carboxyl group.

Interestingly, the three residues (Phe91, Leu419, and Phe327*) located at the active site entrance in YgjG are not conserved; thus, the differences in substrate specificities among the class III aminotransferases might mainly arise from the residues at the active site entrances. The YgjG Phe91 residue is substituted by a tyrosine in AcOAT and OAT and by isoleucine in GABA-AT. The YgjG Phe327* is replaced by a glycine in AcOAT, OAT and GABA-AT. The leucine residue that correponds to YgjG Leu419 was not observed in AcOAT, OAT and GABA-AT. As mentioned above, the bulky phenyl group at residue 327* and the Leu419 side chain protruded into the substrate binding cavity, decreasing the substrate-binding cleft entrance. YgjG has a narrower entrance to the active-site cavity, while other aminotransferases have a wider entrance and narrow towards the PLP cofactor, as shown in Figure 6C. Furthermore, the YgjG substrate-binding site entrance was relatively more hydrophobic than in other aminotransferases. Taken together, the size and charge distribution of the substrate-binding cleft might provide information on the different substrate specificities of class III aminotransferases.

In conclusion, we solved the crystal structure of YgjG from *E. coli*, which is the first structure of an aminotransferase that uses polyamines, including putrescine, as a substrate. When the YgjG structure was compared with other class III aminotransferases, YgjG exhibited an additional N-terminal helical structure, which partially contributes to dimer formation. In addition, the smaller and relatively more hydrophobic active site entrance in YgjG compared with other class III aminotransferases could be the basis for its preference of the primary diamine substrate of putrescine. Our study will contribute to a better understanding putrescine regulation in cells and the different substrate specificities among aminotransferases.

## Supporting Information

Figure S1 **The two half reactions catalyzed by YgjG.**
(DOCX)

Table S1 **Intermolecular polar contacts between subunits of YgjG.**
(DOCX)

Table S2 **Surface area of the dimer interface in YgjG and its structural homologs.**
(DOCX)
