# Supplementary figures and images for: Structure of Putrescine Aminotransferase from Escherichia coli Provides Insights into the Substrate Specificity among Class III Aminotransferases
Source: PLoS One. 2014 Nov 25;9(11):e113212. doi: 10.1371/journal.pone.0113212 (PMC4244111; doi:10.1371/journal.pone.0113212)

**Figure S1. The two half reactions catalyzed by YgjG.**


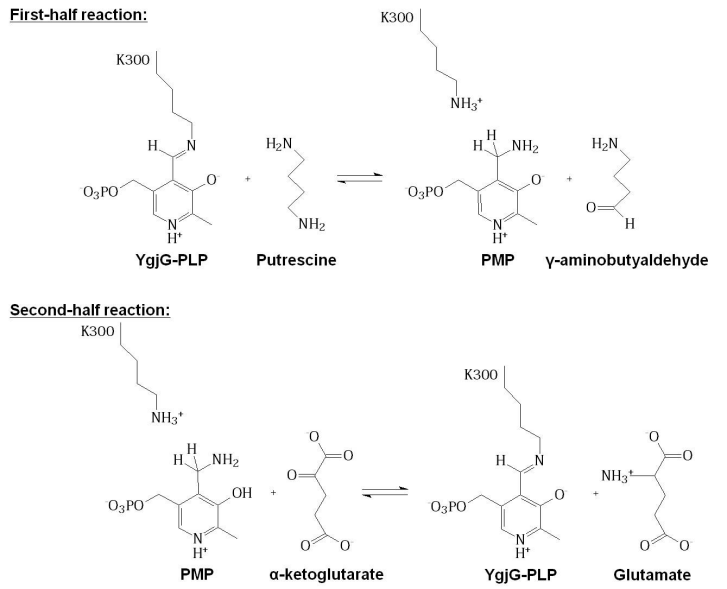

Supplement: Figure S1 — The two half reactions catalyzed by YgjG. (DOCX) [file pone.0113212.s001.docx]
