# Supplementary material for: Structure of Putrescine Aminotransferase from Escherichia coli Provides Insights into the Substrate Specificity among Class III Aminotransferases
Source: PLoS One. 2014 Nov 25;9(11):e113212. doi: 10.1371/journal.pone.0113212 (PMC4244111; doi:10.1371/journal.pone.0113212)
Supplement: Table S1 — Intermolecular polar contacts between subunits of YgjG. (DOCX) [file pone.0113212.s002.docx]

**Table S1. Intermolecular polar contacts between subunits of YgjG**

|  |  | |  | |  | | | |  |  | |  | |  | |  | |
| --- | --- | --- | --- | --- | --- | --- | --- | --- | --- | --- | --- | --- | --- | --- | --- | --- | --- |
| No. | Chain A | | Chain B | | Distance (Å) | | | |  | No. | | Chain A | | Chain B | | Distance (Å) | |
| **H-bonds** | |  | |  | | |  |  | | |  | |  | |  | |  |
| 1 | Leu11 [O] | | Asn348 [ND2] | | | 3.86 | | |  | 31 | | Lys113 [NZ] | | Leu20 [O] | | 3.30 | |
| 2 | Ser14 [OG] | | Asn348 [ND2] | | | 2.95 | | |  | 32 | | Lys113 [NZ] | | Ile21 [O] | | 2.60 | |
| 3 | Leu20 [O] | | Lys113 [NZ] | | | 3.21 | | |  | 33 | | Lys113 [NZ] | | Lys23 [O] | | 2.97 | |
| 4 | Ile21 [O] | | Lys113 [NZ] | | | 2.71 | | |  | 34 | | Tyr143 [OH] | | Lys43 [O] | | 2.70 | |
| 5 | Lys43 [O] | | Tyr143 [OH] | | | 2.85 | | |  | 35 | | Lys142 [NZ] | | Glu44 [OE2] | | 3.07 | |
| 6 | Glu44 [OE2] | | Lys142 [NZ] | | | 2.79 | | |  | 36 | | Lys142 [NZ] | | Glu44 [O] | | 2.53 | |
| 7 | Glu44 [O] | | Lys142 [NZ] | | | 2.91 | | |  | 37 | | Ser144 [N] | | His45 [O] | | 2.98 | |
| 8 | His45 [O] | | Ser144 [N] | | | 3.07 | | |  | 38 | | Leu122 [N] | | Ala65 [O] | | 3.25 | |
| 9 | Ala65 [O] | | Leu122 [N] | | | 3.17 | | |  | 39 | | Ser118 [OG] | | Glu67 [OE1] | | 2.54 | |
| 10 | Glu67 [OE1] | | Ser118 [OG] | | | 2.62 | | |  | 40 | | Gln114 [NE2] | | Glu67 [OE2] | | 2.49 | |
| 11 | Glu67 [OE2] | | Gln114 [NE2] | | | 2.45 | | |  | 41 | | Ser118 [N] | | Glu67 [OE2] | | 2.98 | |
| 12 | Glu67 [OE2] | | Ser118 [N] | | | 2.80 | | |  | 42 | | Gln119 [NE2] | | Gly90 [O] | | 2.78 | |
| 13 | Leu111 [O] | | Arg99 [NE] | | | 2.65 | | |  | 43 | | Arg99 [NE] | | Leu111 [O] | | 2.88 | |
| 14 | Ala112 [O] | | Arg99 [NE] | | | 3.06 | | |  | 44 | | Arg24 [NH1] | | Ala112 [O] | | 3.74 | |
| 15 | Lys113 [O] | | Ala70 [N] | | | 2.97 | | |  | 45 | | Ala70 [N] | | Lys113 [O] | | 2.91 | |
| 16 | Glu120 [OE1] | | Arg54 [NH1] | | | 3.20 | | |  | 46 | | Arg54 [NH1] | | Glu120 [OE1] | | 2.74 | |
| 17 | Glu120 [OE1] | | Tyr53 [OH] | | | 3.08 | | |  | 47 | | Tyr53 [OH] | | Glu120 [OE1] | | 3.08 | |
| 18 | Glu120 [OE2] | | Tyr53 [OH] | | | 3.37 | | |  | 48 | | Tyr53 [OH] | | Glu120 [OE2] | | 3.04 | |
| 19 | Leu122 [O] | | Glu67 [N] | | | 2.89 | | |  | 49 | | Glu67 [N] | | Leu122 [O] | | 2.79 | |
| 20 | Thr132 [OG1] | | Ser14 [N] | | | 3.87 | | |  | 50 | | His45 [ND1] | | Lys142 [O] | | 2.96 | |
| 21 | Lys142 [O] | | His45 [ND1] | | | 2.92 | | |  | 51 | | Ser149 [OG] | | Glu152 [OE2] | | 2.63 | |
| 22 | Glu152 [OE2] | | Ser149 [OG] | | | 2.56 | | |  | 52 | | Leu185 [N] | | Glu155 [OE1] | | 2.91 | |
| 23 | Glu155 [OE1] | | Leu185 [N] | | | 2.93 | | |  | 53 | | Lys159 [NZ] | | Lys183 [O] | | 2.93 | |
| 24 | Lys183 [O] | | Lys159 [NZ] | | | 2.75 | | |  | 54 | | Ser166 [OG] | | Pro199 [O] | | 3.31 | |
| 25 | Phe200 [O] | | Lys162 [NZ] | | | 3.20 | | |  | 55 | | Lys162 [NZ] | | Phe200 [O] | | 3.12 | |
| 26 | Pro202 [O] | | Lys162 [NZ] | | | 3.52 | | |  | 56 | | Lys162 [NZ] | | Pro202 [O] | | 3.58 | |
| 27 | Leu322 [O] | | Asn47 [ND2] | | | 3.06 | | |  | 57 | | Asn47 [ND2] | | Leu322 [O] | | 2.88 | |
| 28 | Phe327 [O] | | Lys183 [NZ] | | | 2.48 | | |  | 58 | | Lys183 [NZ] | | Phe327 [O] | | 2.89 | |
| 29 | Thr330 [OG1] | | Lys183 [NZ] | | | 2.41 | | |  | 59 | | Lys183 [NZ] | | Thr330 [OG1] | | 2.39 | |
| 30 | Asn348[ND2] | | Ser14 [OG] | | | 3.11 | | |  | 60 | | Lys 300 [NZ] | | Thr332 [OG1] | | 3.24 | |
|  |  | |  | | |  | | |  |  | |  | |  | |  | |
| **Salt bridges** | |  | |  | | |  |  | | |  | |  | |  | |  |
| 1 | Glu44 [OE2] | | Lys142 [NZ] | | | 2.79 | | |  | 3 | | Lys142 [NZ] | | Glu44 [OE2] | | 3.07 | |
| 2 | Glu120 [OE1] | | Arg54 [NE] | | | 3.20 | | |  | 4 | | Arg54 [NE] | | Glu120 [OE1] | | 2.74 | |
