# Supplementary material for: Structure of Putrescine Aminotransferase from Escherichia coli Provides Insights into the Substrate Specificity among Class III Aminotransferases
Source: PLoS One. 2014 Nov 25;9(11):e113212. doi: 10.1371/journal.pone.0113212 (PMC4244111; doi:10.1371/journal.pone.0113212)
Supplement: Table S2 — Surface area of the dimer interface in YgjG and its structural homologs. (DOCX) [file pone.0113212.s003.docx]

**Table S2. Surface area of the dimer interface in YgjG and its structural homologs**

|  |  |  |  |  |  |  |
| --- | --- | --- | --- | --- | --- | --- |
| Protein | PDB ID | Total surface area (Å^2^) | Interface area (Å^2^) | N_HB_ | N_SB_ | Δ^i^*G* (kcal/mol) |
| YgjG | 4UOY | 20396.6 (100%) | 6166.7 (30.2%) | 60 | 4 | -98.7 |
| AcOAT | 1VEF | 16711.7 (100%) | 4765.9 (28.5%) | 48 | 8 | -59.1 |
| OAT | 2OAT | 18339.4 (100%) | 5100.0 (27.8%) | 69 | 7 | -63.0 |
| GABA-AT | 1SFF | 18366.7 (100%) | 4506.1 (24.5%) | 67 | 13 | -49.5 |
